# Potential of Wormwood and Oak Bark-Based Supplement in Health Improvement of *Nosema ceranae*-Infected Honey Bees

**DOI:** 10.3390/ani14081195

**Published:** 2024-04-16

**Authors:** Uros Glavinic, Nemanja M. Jovanovic, Nina Dominikovic, Nada Lakic, Milivoje Ćosić, Jevrosima Stevanovic, Zoran Stanimirovic

**Affiliations:** 1 Department of Biology, Faculty of Veterinary Medicine, University of Belgrade, Bul. Oslobodjenja 18, 11000 Belgrade, Serbia; uglavinic@vet.bg.ac.rs (U.G.); nina.dominikovic@vet.bg.ac.rs (N.D.); rocky@vet.bg.ac.rs (J.S.); zoran@vet.bg.ac.rs (Z.S.); 2 Department of Parasitology, Faculty of Veterinary Medicine, University of Belgrade, Bul. Oslobodjenja 18, 11000 Belgrade, Serbia; 3 Department of Statistics, Faculty of Agriculture, University of Belgrade, Nemanjina 6, 11080 Belgrade, Serbia; nlakic@agrif.bg.ac.rs; 4 Institute of Forestry, Kneza Viseslava 3, 11000 Belgrade, Serbia; micko.cosic@gmail.com

**Keywords:** honey bee, *Nosema ceranae*, wormwood, oak bark, anti-*Nosema*, oxidative stress

## Abstract

**Simple Summary:**

In this study, we tested the potential of a wormwood and oak bark-based dietary supplement for *Nosema ceranae* infection control and health improvement of infected honey bees. Results revealed a very good anti-*Nosema* effect and, consequently, the reduction of *Nosema*-induced oxidative stress and immunosuppression. Keeping in mind the absence of adequate anti-*Nosema* treatment, this supplement could be of great importance in the beekeeping sector, especially because of its ecologically friendly (plant-based) nature. Motivated by the results of this study, we plan to conduct further experiments to improve knowledge about the effects and mechanisms included in bee health improvement with the tested supplement.

**Abstract:**

*Nosema ceranae*, a microsporidian parasite, as one of the stressors that contribute to honey bee decline, has a significant negative impact on the longevity, productivity, and reproductive capacity of honey bee colonies. There are several different strategies for *Nosema* infection control, including natural-based and antibiotic-based products. In this study, we tested wormwood and oak bark-based supplement “Medenko forte” on survival, *Nosema* infection, oxidative stress, and expression of immune-related genes in artificially *N. ceranae*-infected bees. The results revealed a positive influence on the survival of *Nosema*-infected bees, irrespectively of the moment of supplement application (day 1, day 3, or day 6 after bee emergence), as well as reduction of *Nosema* loads and, consequently, *Nosema*-induced oxidative stress. Supplementation had no negative effects on bee immunity, but better anti-*Nosema* than immune-stimulating effects were affirmed based on expression levels of abaecin, defensin, hymenoptaecin, apidaecin, and vitellogenin genes. In conclusion, the tested supplement “Medenko forte” has great potential in the health protection of *Nosema*-infected bees. However, further investigations need to be performed to elucidate its mechanisms of action.

## 1. Introduction

Microsporidium *Nosema ceranae*, recently redefined as *Vairimorpha* [1], is considered one of the stressors that contribute to honey bee decline [2,3,4,5,6]. Different negative effects of *N. ceranae* on bees have been described. It has been proven to have a significant negative impact on the longevity, productivity, and reproductive capacity of honey bee colonies [7,8]. Infection with *N. ceranae* has negative effects on the metabolism of honey bees, inducing energetic stress and an increase in appetite [9], and as a result, honey and pollen reserves may decrease in such colonies [8]. Moreover, *N. ceranae* infection negatively affects honey bees by reducing olfactory learning and memory [10], causing premature foraging in worker bees [9,11,12], decreasing homing ability [13], and weakening flight ability [14]. Finally, *N. ceranae* leads to suppression of the immune response and increased oxidative stress in bees [15,16,17,18,19].

The negative effect of *N. ceranae* increases when bees are exposed to additional stressors, such as pesticides, other pathogens and parasites, and poor nutrition [4]. The combination of poor nutrition and *Nosema* infection suppresses bees’ immune system [20,21], which can consequently lead to a decrease in bees’ ability to deal with other stressors and, finally, to an increase in mortality rates [4].

For the control of nosemosis, different strategies have been established [22], but there is still no ideal preparation for the treatment of nosemosis. Previously, fumagillin, an antibiotic obtained from the fungus *Aspergillus fumigatus*, was used, but its use was compromised due to the possibility of contamination of bee products. Moreover, some research revealed that fumagillin possesses genotoxic potential [16]. Additionally, there are no registered formulations of fumagillin in Europe. For this reason, the potential of numerous natural products was investigated, but most of them were never used in practice. Good beekeeping practices and biosecurity measures can significantly reduce the possibility of the disease. One of the most important approaches is the application of supplements in the bee diet [4,23]. Many dietary supplements improve honey bee strength, immunity, and performance, thereby contributing to the maintenance of healthy colonies [8,15,19,24,25,26,27,28]. Many studies investigated natural strategies against nosemosis, with plant and algal extracts as well as synthetic vitamin/mineral mixtures giving the best results [29,30,31,32,33,34,35,36,37,38,39,40,41,42,43,44]). These products, administrated to bees before or after the *Nosema* infection, significantly reduced mortality and infection rates [15,16,17,18,19]. It has also been proven that the aqueous extracts of *Agaricus braziliensis* and *A. bisporus* mushrooms positively influence *Nosema*-infected bees, supporting the survival of infected bees [16,17]. Among plant compounds, thymol, oregano, carvacol, reservatol, and garlic extracts showed the potential to decrease *Nosema* loads [45,46,47,48,49]). There are some phyto-pharmacological supplements available on the market (Nozevit and Nozevit plus) that include plant polyphenols, vitamins, minerals, and amino acids [50,51]. These supplements reduced *Nosema* infection, and the effect was described as the formation of a mucous layer on the midgut’s epithelial cells, which prevents parasites from entering farther [50,51]. Herbal supplements NOZEMAT HERB^®^, NOZEMAT HERB PLUS^®^ [25], and B+ [8] also showed a positive influence on the general development of honey bee colonies and significant biological activity against *N. ceranae* in infected apiaries [8,25].

Bearing that in mind, we have conducted a cage experiment to test a dietary supplement commercially available under the name “Medenko forte”, a blend of oak bark, sage, and wormwood extracts, analyzing (i) bee survival, (ii) *Nosema* development dynamics, (iii) oxidative stress parameters, and (iv) expression levels of genes important for immunity.

## 2. Materials and Methods

### 2.1. Tested Supplement

A dietary supplement by the brand name “Medenko forte” (Certificate No. 323-07-2337/14-05, Matex, Šabac, Serbia) was tested. This supplement is composed of standardized liquid extract (1:2) sage (*Salvia officinalis*) leaf 10%, liquid extract (1:2) oak bark 10%, and tincture (1:2) wormwood (*Artemisia absinthium*) herb 10%. The feeding solution was prepared according to the manufacturer’s instructions: 100 mL of the supplement was dissolved in 900 mL of sugar (sucrose) syrup 50% (*w*/*v*).

### 2.2. Design of Laboratory Experiment

The experiment was carried out in the laboratory of the Department of Biology, Faculty of Veterinary Medicine, University of Belgrade. Frames with sealed brood prior to emergence were taken from the experimental apiary of the Faculty of Veterinary Medicine, University of Belgrade. Frames were taken from the five randomly chosen bee colonies without signs of diseases of adult bees or brood and were placed into the incubator at 34 ± 1 °C and 66 ± 1% relative humidity. After 24 h, 80 randomly newly emerged bees were placed in each cage, designed specifically for this kind of experiment [15]. Briefly, a plastic jar punctured to let air in was placed with the top (lid) down, and a plastic strainer was placed in the middle of the lid so bees could collect the syrup from the tiny petri dish underneath. The whole experiment was repeated, and the results were merged into a single dataset.

Six groups were established, and each group comprised three cages/replicates (Table 1); all were fed a 50% (*w*/*v*) sucrose (Centrohem, Stara Pazova, Serbia) solution. The first group received supplement “Medenko forte” from the 1st day (S); three groups were *Nosema*-infected and received supplement “Medenko forte” starting from day 1 after emergence (I-S1), day 3 (I-S3), and day 6 (I-S6); the remaining two groups were controls (one *N. ceranae*-infected (I) and another non-infected (NI)). From each cage, 15 bees were sampled for gene expression measurements and 15 bees for oxidative stress analyses (5 bees for each of three sampling moments); 10 bees on each sampling day (30 in total) were collected for *Nosema* spore counting while the rest of 20 bees served for mortality monitoring. The dead bees were counted and taken out of the cages every day.

### 2.3. Inoculum Preparation and Experimental Infection

The infection was performed according to a previously established methodology [15]. Briefly, forager honey bees were sampled from *Nosema*-infected colonies. The abdomens of obtained foragers were individually crushed in distilled water. Freshly prepared spore suspension with 99% viability, assessed with 4% trypan blue (Sigma–Aldrich, Steinheim, Germany), was quantified using a hemocytometer according to Cantwell [52] and mixed with sugar solution to obtain a final concentration of 1 × 10^6^ spores/mL. Before experimental infection, *N. ceranae* species had been confirmed using the PCR technique as described in Stevanovic et al. [53], and the absence of other bee pathogens such as *N. apis*, *Lotmaria passim*, ABPV, CBPV, and DWV was confirmed by PCR and RT-PCR with specific primers [4,8]. In *Nosema*-infected groups (I, I-S1, I-S3, and I-S6) on day 3, we performed individual inoculation of bees with *N. ceranae* spores, according to BEEBOOK [54] and our experimental design (Table 1). Two hours before the infection, the food was taken out of the cages to starve the bees and improve their intake of the inoculum.

### 2.4. Nosema ceranae Spores Counting

The number of *N. ceranae* spores was determined according to the methodology described in Glavinic et al. [16]. Briefly, the abdomen of each bee was placed in a 1.5 mL tube and homogenized in 1 mL of distilled water in Tissue Lyser II (Qiagen, Hilden, Germany) for 1 min at 25 Hz. The number of *N. ceranae* spores was determined using a hemocytometer and methodology described by Cantwell [52]. To count the spores, the improved *Neubauer* hemocytometer with cover-slip was used. The sample was added to the hemocytometer, and the spores settled at the chamber’s bottom after a few minutes. An optical microscope with a magnification of ×400 was used to visualize and count the spores. The number of spores in five squares was determined. Only those spores that crossed the square’s upper and left edges (not its bottom or right edges) were counted when a spore fell over its edge. The sum of the spore counts from five squares was then multiplied by 50.000 to obtain the number of spores per bee [15].

### 2.5. Analyses of Oxidative Stress Parameters

The activities of the antioxidative enzymes superoxide dismutase (SOD), catalase (CAT), and glutathione S-transferase (GST), as well as the concentrations of malondialdehyde (MDA), were assessed through spectrophotometric analyses using a UV/VIS Spectrophotometer BK-36 S390 (Biobase, Jinan, China), as detailed in previous studies [16,18,27]. The analysis involved examining pools of five bees that were gathered from each cage on three sampling time points (6, 9, and 15).

Homogenates of whole bees were made in a mortar with liquid nitrogen and tris-HCl buffer that had been pH-adjusted to 7.4. Following a 10-min centrifugation at 10,000× *g*, the supernatant was poured off and stored at −20 °C until needed. The units of activity per milligram of protein (U/mg) for the enzymes’ specific activity and nmol/mg of protein for the MDA concentration were calculated and presented.

Superoxide dismutase (SOD) activity was measured kinetically using a change in absorbance over time at a wavelength of 480 nm. The reaction mixture was incubated at 30 °C for three minutes after the addition of adrenaline. The amount of enzyme required to reduce the rate of autoxidation by 50% in an alkaline environment was established as the unit of enzyme activity.

The spectrophotometric observation of H_2_O_2_ breakdown at 240 nm serves as the foundation for the determination of catalase (CAT) activity in bee homogenates. The activity is tracked as the absorbance at the specified wavelength decreases.

1-chloro-2, 4-dinitrobenzene (CDNB) served as the substrate for the glutathione-S-transferase (GST) activity measurement. The process relies on CDNB’s capacity to combine with glutathione, which GST catalyzes, to form a complex. The molar extinction coefficient for CDNB is used to calculate the velocity of complex formation. The number of nM of glutathione oxidized per minute is the definition of specific activity. The method for quantifying the amount of malondialdehyde (MDA) in homogenates is based on the fact that MDA, a particular byproduct of lipid peroxidation, combines with thiobarbituric acid (TBA) to create the colorful MDA-TBARS complex, which has a maximum absorbance at 535 nm. A molar extinction coefficient is used to compute the concentration.

### 2.6. Gene Expression Analyses

The total RNA was extracted with Quick-RNA MiniPrep Kit (Zymo Research, Irvine, CA, USA). Each single bee was added to a 1.5 mL tube with 500 μL of Genomic Lysis Buffer. Homogenization was performed in a TissueLyser II (Qiagen, Hilden, Germany) for 1 min at 25 Hz using a 3 mm tungsten carbide bead (Qiagen, Hilden, Germany). Other extraction steps were performed according to the manufacturer’s instructions. For cDNA synthesis, 1000 ng of RNA per sample was immediately reverse-transcribed using RevertAid™First Strand cDNA Synthesis Kit (Thermo Fisher Scientific, Vilnius, Lithuania).

The expression level of genes was determined using the SYBR Green method (protocol according to KAPA SYBR FAST qPCR Kit Master Mix (2X) Universal, KAPA Biosciences), applying 2^−ΔΔCt^ method [55], modified as described in our previous study [16], and 20 μL reactions were processed in “Rotor-Gene Q 5plex” (Qiagen, Hilden, Germany). The amplification was performed according to the following protocol: 95 C for 2 min followed by 40 cycles of 95 °C for 5 s and annealing temperatures for 30 s. Transcript levels of genes for *abaecin*, *apidaecin*, *defensin*, *hymenoptaecin,* and *vitellogenin* were determined. The primers for target genes and an internal control gene are described in our previous research [16]. The median value of the NI group served as a calibrator, while *b-actin* was used as an internal control gene [15].

### 2.7. Statistical Analyses

The dynamics of survival in groups of bees is shown by the Kaplan–Meier survival function. The difference in survival dynamics between the two groups was examined by the log-rank test.

There was heterogeneity in the gene expression data and the number of *Nosema* spores in the experimental groups. In order to test the hypothesis on the equality of medians among three or more groups as well as at the three-time points of sampling, the Kruskal–Wallis test was utilized. In addition, the significance of the difference between pairs was ascertained using the Mann–Whitney U test. The semi-logarithmic diagram is utilized to visually represent the relationship between the mRNA levels in the groups.

Oxidative stress values in the samples showed homogeneity. All oxidative stress parameters were compared between experimental groups on the same day of sampling using discriminant analysis. For each oxidative stress measure, the ratio of three or more arithmetic means was tested using ANOVA, and the ratio of the means of two samples was tested using Tukey’s test.

All conclusions use a 0.05 level of significance. Experimental data were analyzed using Statistica Software (Version 10, StatSoft, Inc., Tulsa, OK, USA).

## 3. Results

### 3.1. Bee Survival

**Figure 1:**
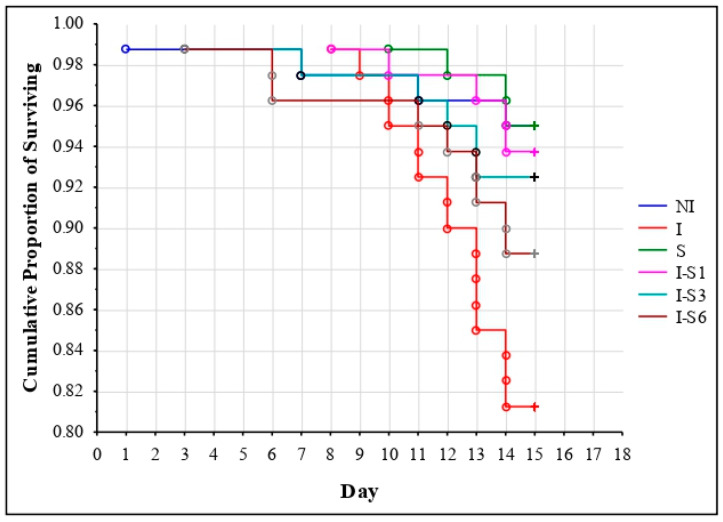
Survival of bees in the group treated with supplement (S), non-infected control (NI), *N. ceranae*-infected control (I), and groups infected and treated with the supplement from day 1 (I-S1), day 3 (I-S3), and day 6 (I-S6). Group names are presented in Table 1.

When observed all groups simultaneously, significant differences in survival dynamics were detected (χ^2^ = 13.663; *p* = 0.018). The log-rank test showed significantly (*p* = 0.008) better survival of bees from the NI (non-infected) control group compared to the *Nosema*-infected control (I). Additionally, better survival was recorded in all groups treated with supplement (S, I-S1, and I-S3) compared to the I group (*p* = 0.007, *p* = 0.017, and *p* = 0.039, respectively) with the exception of the I-S6 group (*p* = 0.194) (Figure 1).

### 3.2. Number of Nosema ceranae Spores

**Figure 2:**
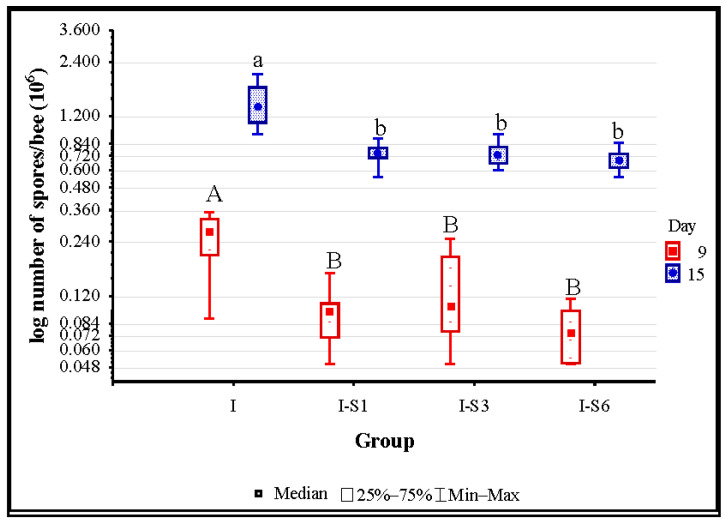
*Nosema* spore loads in infected control (I) and groups infected and treated with the supplement from day 1 (I-S1), day 3 (I-S3), and day 6 (I-S6). Different letters denote significant differences between the groups. Group names are presented in Table 1.

Bee samples collected from uninfected groups (NI and S) exhibited no evidence of spore presence from this particular endoparasite in all the three sampling moments (days 6, 9, and 15). Additionally, the absence of *Nosema* spores was confirmed in all experimental groups on day 6. On days 9 and 15, according to the Kruskal–Wallis test, a significant difference in spore count was present among infected groups (*p* < 0.001). On day 9, the highest spore load was detected in group I and it was significantly (according to the Mann–Whitney U test) higher than in other infected groups: I-S1 (*p* = 0.001), I-S3 (*p* = 0.008), and I-S6 (*p* < 0.001). Similarly, on day 15, group I was more loaded with *Nosema* spores than groups I-S1 (*p* < 0.001), I-S3 (*p* < 0.001), and I-S6 (*p* < 0.001) (Figure 2).

### 3.3. Oxidative Stress Parameters

**Figure 3:**
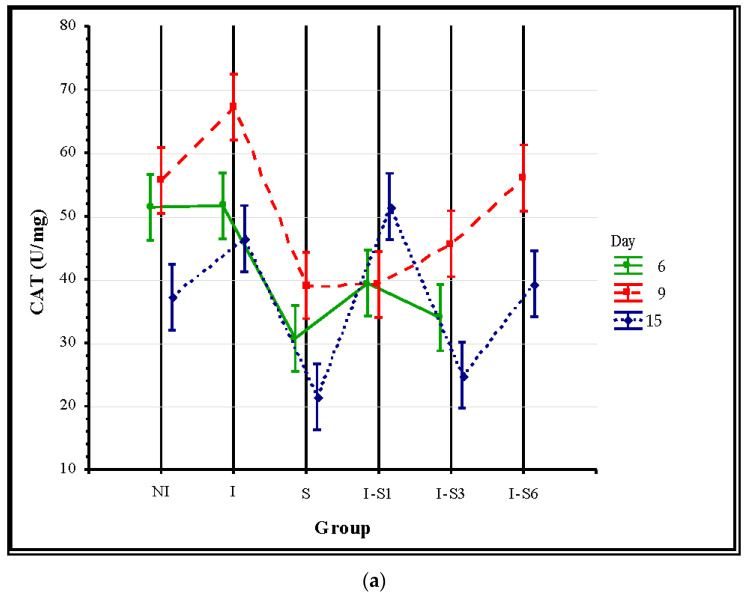

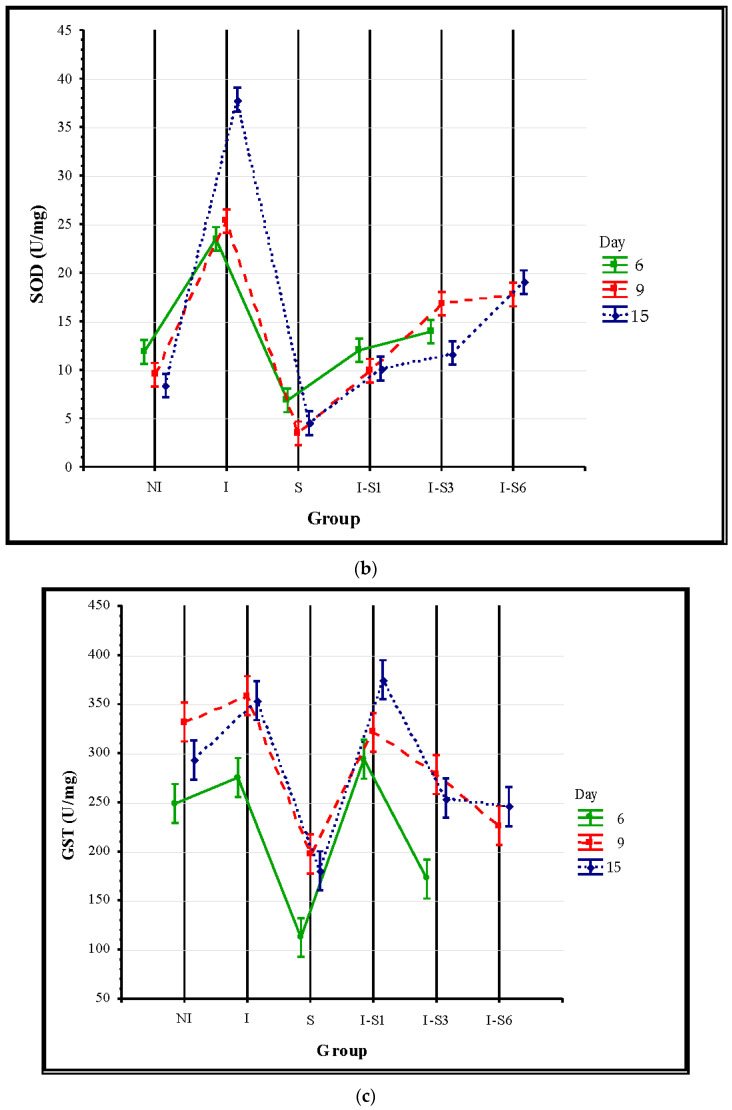

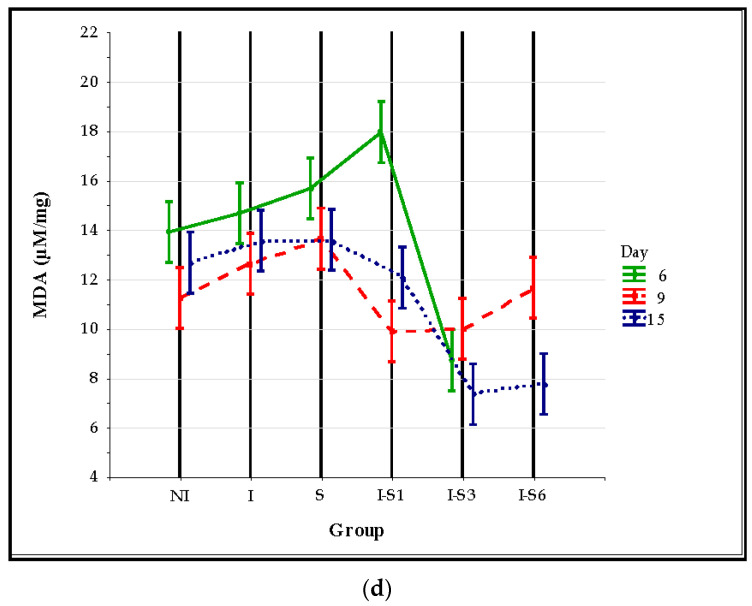
Mean values for (**a**) catalase—CAT, (**b**) superoxide dismutase—SOD and (**c**) glutathione S-transferase—GST activities and (**d**) malondialdehyde—MDA concentration. Group treated with supplement (S), non-infected control (NI), *N. ceranae*-infected control (I), and groups infected and treated with the supplement from day 1 (I-S1), day 3 (I-S3), and day 6 (I-S6). Group names are presented in Table 1.

The results of the discriminant analysis showed that on days 6, 9, and 15, the differences between all analyzed groups, as well as between the pairs of the two groups, were statistically significant (*p* < 0.05) when all four parameters of oxidative stress were observed simultaneously. At the time of bee sampling (days 6, 9, and 15), ANOVA revealed significant differences (*p* < 0.001) in enzyme activity (SOD, CAS, and GST) and MDA concentration among all the groups. The most significant differences were identified on day 15. According to the Tukey’s test on day 15, SOD enzyme activity was not significantly different only in group I-S1 compared to NI (*p* = 0.487) and I-S3 (*p* = 0.579). Catalase (CAT) activity was significantly lower (*p* < 0.05) in group S compared to all the other groups except I-S3. Moreover, it was significantly higher in group I-S1 compared to groups NI and I-S3, and it was significantly lower in group I-S3 compared to I and I-S6. (Figure 3). By analyzing the activity of the GST enzyme, significantly higher (*p* < 0.001) values were found in groups I and I-S1 compared to the other experimental groups, while there were no significant differences only between groups I-S3 and I-S6 (*p* = 0.535). The concentration of MDA was not different when compared between groups I-S3 and I-S6 (*p* = 0.998) but was significantly lower (*p* < 0.001) in each of them when compared to all other experimental groups (Figure 3).

### 3.4. Gene Expression

**Figure 4:**
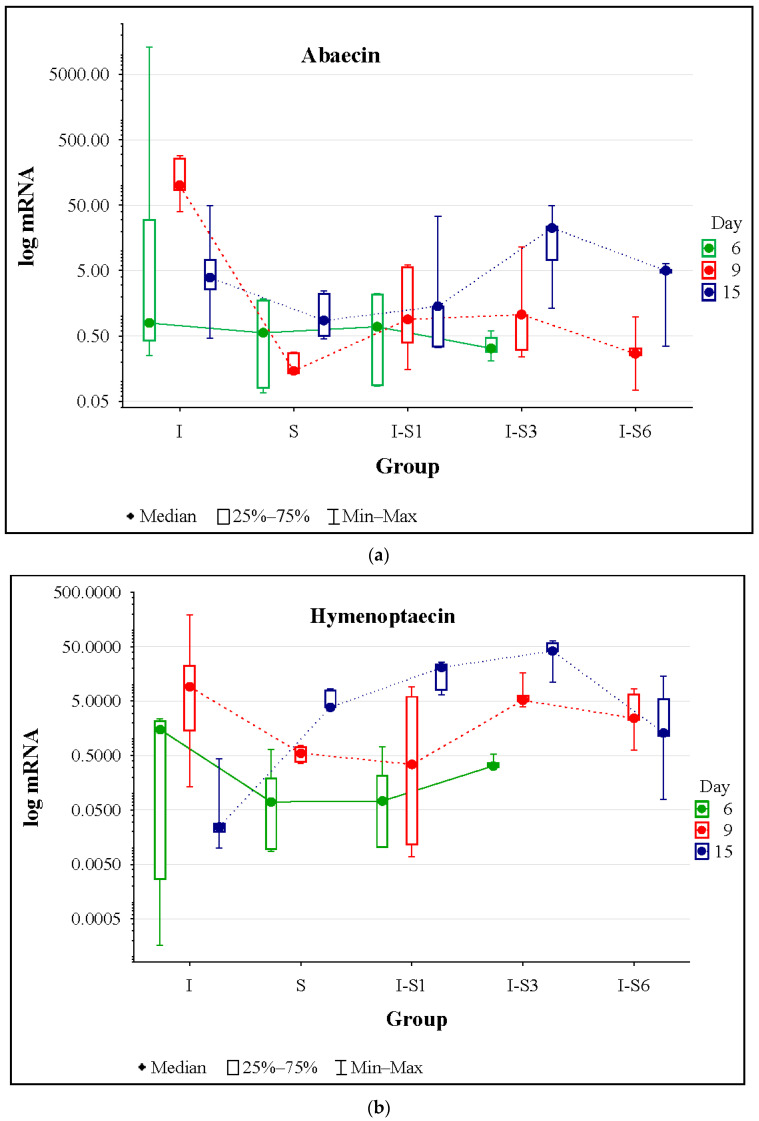

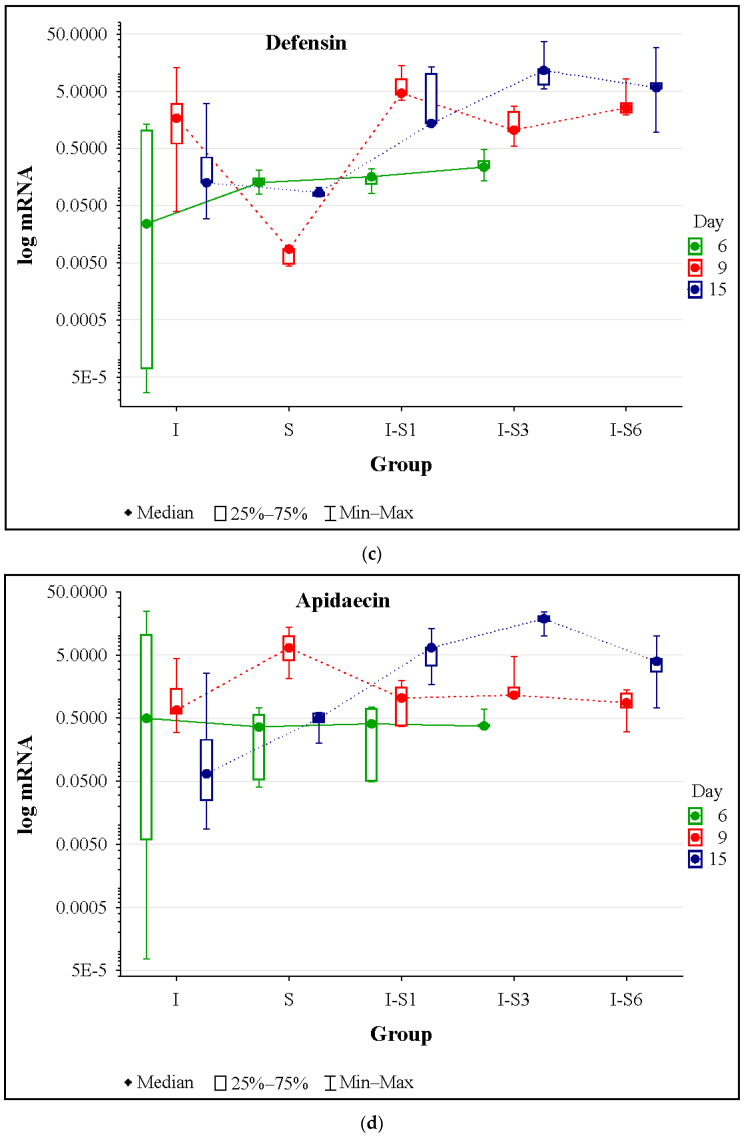

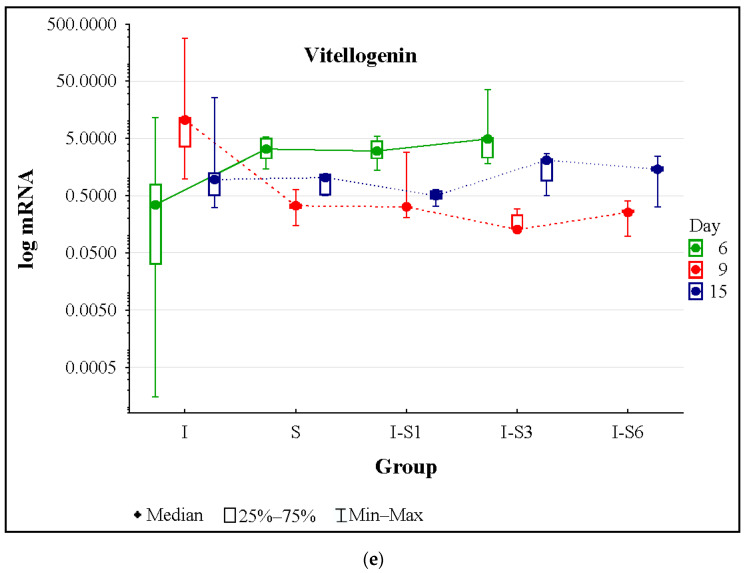
Expression levels of immune-related genes: abaecin (**a**), hymenoptaecin (**b**), defensin (**c**), apidaecin (**d**), and vitellogenin (**e**) at different time points in experimental groups. Group treated with supplement (S), *N. ceranae*-infected control (I), and groups infected and treated with the supplement from day 1 (I-S1), day 3 (I-S3), and day 6 (I-S6). Group names are presented in Table 1.

According to the Kruskal–Wallis test, on day 6, there were no significant differences (*p* > 0.05) in expression levels of all monitored genes (Figure 4 and Supplementary Figure S1). Levels of gene expression in bees collected on day 9 were significantly different (*p* < 0.05) and without a clear pattern, except expression levels for hymenoptaecin (*p* = 0.0877). However, it is interesting that in group S, which received the supplement without infection, levels of gene expression for abaecin and defensin were lower than in other groups, even lower than in the infected control (I) group (Figure 4 and Supplementary Figure S1). According to the experimental design and dynamics of *Nosema* infection, the most important gene expression results were those recorded on day 15 (Figure 4 and Supplementary Figure S1). According to Mann–Whitney U Test, on that day, levels of hymenoptaecin gene transcripts were lowest in group I (Figure 4 and Supplementary Figure S1) and significantly lower than in all other groups (*p* < 0.05). However, vitellogenin gene transcript levels were highest in group I. When it comes to the expression levels of defensin and apidaecin genes, values in groups I and S were significantly lower (*p* < 0.05) than in other groups.

## 4. Discussion

The beneficial effects of natural compounds on the lifespan of *Nosema*-infected honey bees have already been proven [45,46]. In our research, the natural-based tested supplement “Medenko forte” also showed a positive effect on bee survival rate since it was significantly higher in groups that received the tested supplement irrespectively of the day when the test supplement was introduced into the food: before *Nosema* infection (I-S1), at the same time with infection (I-S3), and even after infection (I-S6), compared to the infected group. The positive effect of the tested supplement on bee survival was also confirmed by the survival rates registered in group S, where the supplement did not have negative impacts on the survival of non-infected bees. In previous studies, different types of diets were tested for their effect on *Nosema*-infected bees. It has been proven that some commercially available supplements like B+ [19] and “BEEWELL AminoPlus” [15] showed a positive effect on the survival of bees infected with *Nosema* as well as the mushroom extracts of *Agaricus blazei* and *A. bisporus* [16,17] and various plant extracts either aqueous extracts of plants such those of *Thymus vulgaris*, *Salvia officinalis*, *Plantago lanceolata* and *Rumex acetosella* [56], or alcoholic extracts such those of *Rosmarinus officinalis* and *Ugni molinae* [23].

In this study, the number of *Nosema* spores did not significantly differ between groups fed with the supplement “Medenko forte” and infected with *Nosema* (groups I-S1, I-S3, and I-S6), either on day 9 or 15. This indicates that the supplement had the same beneficial impact when administered prior to the infection (I-S1), simultaneously with infection (I-S3) and after infection (I-S6). The importance of this result is essential, considering the persistent presence of *Nosema* infection in the hive and the permanent exposure of the bee population to the pathogen [57,58]. The anti-*Nosema* effect of the oak bark extract rich with tannins (which is present in the supplement “Medenko forte”) has also been shown in previous studies [50,51,59]. These studies explained the effect by stimulating the production and secretion of mucus in epithelial cells, which form a protective layer with a peritrophic membrane [59]. This reduces the number of infected cells in the bee’s midgut and decreases the level of *Nosema* infection in bees, as well as its damaging effect. In addition to oak bark extract, “Medenko forte” also contains the extract of wormwood, which also has been demonstrated to possess an anti-*Nosema* effect [60,61,62,63,64].

The findings of our research are in accordance with previous studies that have confirmed the anti-*Nosema* effects of various herbal extracts and supplements. Chaimanee et al. [23] demonstrated that plant extracts made from *Annona squamosa*, *Ocimum basilicum*, *Psidium guajava,* and *Syzygium jambos* possess a strong anti-microsporidian activity and inhibit the development of *N. ceranae* spores, with similar efficacy to fumagillin. In another recent study conducted by Özkırım et al. [56], the results showed that herbal extract mixture containing *Rumex acetosella*, *Achillea millefolium*, *Plantago lanceolata*, *Salvia officinalis*, *Thymus vulgaris*, *Rosmarinus officinalis,* and *Laurus nobilis* was more effective than fumagillin. Similarly, thymol was shown to possess an anti-*Nosema* effect in several studies [18,45,65]. In our previous studies, the anti-*Nosema* effect was achieved using commercial supplements such as “B+” [19] and “BEEWELL AminoPlus” [15] in laboratory conditions, but also in hive experiments [8,27]. Anti-*Nosema* effects of mushroom species *Agaricus bisporus* and *A. blazei* were observed without any side effects when preventively applied [16,17].

The activities of all antioxidant enzymes (CAT, GST, and SOD) in bees that received the supplement “Medenko forte” were in almost all cases lower compared to the control infected group (I), especially at the end of the experiment (day 15), which can be interpreted as the potential of the tested supplement in the prevention of oxidative stress. Similar results were reported in our previous laboratory experiment of other plant-based supplement B+ [19]. This study showed that the activity of CAT and GST, as well as MDA concentrations, in the group that received the supplement preventively (I-S1) were similar or even higher than those in the infected control group (I), indicating similar or increased levels of oxidative stress in certain situations. In other words, the anti-*Nosema* effect of the supplement is preferable to its antioxidant effect, as demonstrated by these results. It is known that oak bark extract, which is one of the main ingredients of “Medenko forte”, is rich in phenolic compounds called tannins [59]. Phenolics with a high molecular mass possess an adequate quantity of hydroxy groups that create complexes with proteins, cellulose, and certain minerals [59], which can cause oxidative stress.

Bees that received the supplement “Medenko forte” experienced increased expression of most monitored genes compared to the infected group (I). This is in line with other molecular-genetic research that confirmed the negative impact of *N. ceranae* infection on the health and lifespan of bees through the influence on their genes important for metabolism, nutrition, hormonal regulation, immunity, energy homeostasis, and behavioral maturation [66,67,68,69,70,71,72,73]. The authors found a connection between metabolic disorders, energetic, hormonal, nutritional, and oxidative stresses, and immunosuppression [4,74]. On the contrary, most of the monitored genes in group S had lower expression compared to the groups that received the supplement and were *Nosema*-infected (I-S1, I-S3, and IS6). The gene expression levels in group S were between the levels recorded in I-S1, I-S3, and IS6 and those in group I (infected control). This finding indicates that the anti-*Nosema* effect of the tested supplement was greater than its immunostimulant effect. The anti-*Nosema* effect is also supported by the results of the *N. ceranae* spore load at the end of the experiment, as well as the results of the levels of oxidative stress. In our previous studies, some natural-based products revealed better immunostimulatory effects, e.g., extracts of *Agaricus* sp. mushrooms [16,17] and BEEWELL AminoPlus [15]. However, some other supplements, similar to “Medenko forte”, showed a higher anti-*Nosema* mode of action, such as thymol [18] and the well-known drug fumagillin [16].

## 5. Conclusions

Our findings indicate that the tested wormwood and oak bark-based supplement in laboratory conditions shows a positive influence on bee survival in *Nosema*-infected groups, irrespectively of the moment of supplement application (day 1, day 3, or day 6 after bee emergence), since in all these groups *Nosema* spore loads were reduced comparing to infected control. However, the expression of analyzed immune-related genes in these groups was higher than in the *Nosema*-infected control group but not consistently higher in treatment control group S, which suggests the absence of a pure immunostimulating effect of tested preparation. In most cases, the oxidative stress was reduced in infected and treated groups, suggesting that supplement application reduced *Nosema*-induced oxidative stress through the abovementioned anti-*Nosema* effect. Further investigations, including those on honey bee cell lines, may help in elucidating the mechanism of action of the tested supplement.

## Figures and Tables

**Table 1:** Experimental design.

Groups	Day after Emergence, When the Test Supplement Was Introduced into the Food	Day after Emergence, When the Infection with *Nosema ceranae* was Conducted	Sampling Day		
NI	-	-	6	9	15
I	-	3	6	9	15
S	1	-	6	9	15
I-S1	1	3	6	9	15
I-S3	3	3	6	9	15
I-S6	6	3	-	9	15

## Data Availability

The data presented in this study are available on request from the corresponding author. The data are not publicly available due to the excessive data size.

## Supplementary Materials

The following supporting information can be downloaded at https://www.mdpi.com/article/10.3390/ani14081195/s1, Figure S1: Medians of relative expression ratios for immune-related genes (*abaecin*, *hymenoptaecin*, *defensin*, *apidaecin,* and *vitellogenin*) in experimental groups.
