# Supplementary figures and images for: Potential of Wormwood and Oak Bark-Based Supplement in Health Improvement of Nosema ceranae-Infected Honey Bees
Source: Animals (Basel). 2024 Apr 16;14(8):1195. doi: 10.3390/ani14081195 (PMC11047348; doi:10.3390/ani14081195)

| Day | Group | Abaecin | Hymenoptecin | Defensin | Apidecin | Vitelogenin |
|-----|-------|---------|--------------|----------|----------|-------------|
| 6   | I     |         |              |          |          |             |
|     | S     |         |              |          |          |             |
|     | I-S1  |         |              |          |          |             |
|     | I-S3  |         |              |          |          |             |
| 9   | I     |         |              |          |          |             |
|     | S     |         |              |          |          |             |
|     | I-S1  |         |              |          |          |             |
|     | I-S3  |         |              |          |          |             |
|     | I-S6  |         |              |          |          |             |
| 15  | I     |         |              |          |          |             |
|     | S     |         |              |          |          |             |
|     | I-S1  |         |              |          |          |             |
|     | I-S3  |         |              |          |          |             |
|     | I-S6  |         |              |          |          |             |

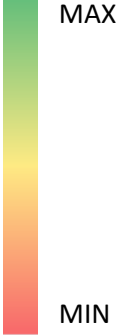

Supplement: Supplementary file 1 [file animals-14-01195-s001.zip › animals-2925951-supplementary.pdf]
